# Efficacy and Safety of Conventional Versus Super-bioavailable Itraconazole in Dermatophytic Infections: A Prospective Randomized Comparative Study

**DOI:** 10.7759/cureus.103654

**Published:** 2026-02-15

**Authors:** Sakshi Sahni, K Geetha, Amrita Upadhyaya, Sana Islahi

**Affiliations:** 1 Department of Dermatology, All India Institute of Medical Sciences, Raebareli, IND; 2 Department of Dermatology, ESIC Medical College and Hospital, Chennai, IND; 3 Department of Dermatology, Indira Gandhi Super-Speciality Centre, Raebareli, IND; 4 Department of Microbiology, All India Institute of Medical Sciences, Raebareli, IND

**Keywords:** dermatophytosis, mycological cure, pharmacokinetics, recalcitrant, super-bioavailable itraconazole

## Abstract

Background: Dermatophytosis has emerged as a therapeutic challenge in recent years due to suboptimal response to conventional antifungal therapy and frequent relapses. While conventional itraconazole (C-ITZ) remains a mainstay of treatment, super-bioavailable itraconazole (SB-ITZ) has emerged as a promising alternative with improved pharmacokinetics. This study compares the efficacy and safety of C-ITZ and SB-ITZ in the management of dermatophytosis.

Materials and methods: This prospective, randomized, open-label, parallel-group comparative study included 108 patients with clinically and mycologically confirmed dermatophytosis involving >20% body surface area. Participants were randomized to receive either C-ITZ 100 mg twice daily or SB-ITZ 50 mg twice daily for four to six weeks. Clinical, mycological, and complete cure rates were assessed at four and six weeks. Safety profile and relapse rates were also evaluated.

Results: Complete cure rates were low at four weeks in both groups. At six weeks, most patients in both groups achieved clinical and mycological cures. Although cure rates were numerically higher in the SB-ITZ group, no statistically significant difference was observed between the two formulations overall. In recalcitrant dermatophytosis, SB-ITZ demonstrated a significantly higher clinical cure rate at six weeks (p = 0.029), while mycological cure and relapse rates were similar. Adverse events were more frequent in the C-ITZ group.

Conclusions: SB-ITZ and C-ITZ showed comparable overall efficacy in dermatophytosis. SB-ITZ demonstrated a modest clinical advantage in recalcitrant cases with better tolerability. Treatment duration, adherence, and hygiene practices remain key determinants of long-term outcomes.

## Introduction

Dermatophytosis ranks among the most prevalent superficial fungal infections worldwide, with estimates indicating that nearly 20-25% (one-fifth) of the global population may be affected at any given time. Recent Indian data indicate a rising burden of chronic and recurrent dermatophytosis, reflecting a growing therapeutic challenge [1].

Treatment of dermatophytosis has evolved significantly over the past decades, shifting from toxic topical agents to systemic antifungal agents. Griseofulvin, introduced in 1958, was the first effective oral therapy, followed by azoles such as ketoconazole, fluconazole, and itraconazole [2]. Despite these advances, management of dermatophytosis has become increasingly challenging due to high relapse rates, prolonged therapy requirements, and the indiscriminate use of topical corticosteroid-antifungal combinations [3]. Itraconazole remains a cornerstone systemic antifungal, but conventional itraconazole (C-ITZ) therapy in India often yields suboptimal clinical responses. Notably, studies have not demonstrated significant drug-resistance mutations or increased virulence in circulating dermatophytes, suggesting that treatment failure may be influenced more by pharmacokinetic variability than by fungal factors [4].

C-ITZ exhibits variable bioavailability, affected by gastric pH, food intake, and interindividual differences, which may compromise treatment efficacy. To address these limitations, a novel formulation, super-bioavailable itraconazole (SB-ITZ), was developed using a solid dispersion system, enhancing absorption and providing more predictable serum concentrations [5]. Unlike conventional pellets, SB-ITZ delivers the drug directly to the small intestine, thereby improving both bioavailability and potential clinical effectiveness. Pharmacokinetic studies indicate that SB-ITZ has a relative bioavailability of approximately 173% compared with C-ITZ, meaning that a 58 mg dose of SB-ITZ yields systemic drug exposure equivalent to that of 100 mg of C-ITZ. Consequently, both the 50 mg and 65 mg SB-ITZ formulations provide drug exposure comparable to the standard 100 mg C-ITZ dose [6]. Based on these data, the 50 mg formulation of SB-ITZ was selected for our study to achieve effective systemic exposure while maintaining a lower pill burden for patients.

Early Indian studies comparing C-ITZ and SB-ITZ, beginning in 2021 [7], suggest improved therapeutic outcomes with SB-ITZ. However, these studies were limited by small sample sizes, retrospective designs, and short follow-up periods. Given the current dermatophytosis epidemic in India and the limitations of conventional therapy, there is a pressing need for prospective studies to guide treatment decisions. This study aims to compare the efficacy and safety of conventional versus SB-ITZ in a randomized, prospective design, with a 12-week follow-up to assess clinical response, relapse, and adverse events, thereby providing robust data to inform evidence-based management.

Aims and objectives

The present study aimed to generate robust clinical evidence on the efficacy and safety of SB-ITZ in the treatment of dermatophytosis. An additional aim was to evaluate the therapeutic efficacy of SB-ITZ in adolescents aged 15-18 years, a group that remains underrepresented in antifungal research. The primary objective was to compare the clinical, mycological, and complete-cure rates of C-ITZ and SB-ITZ at four and six weeks. Secondary objectives included assessing safety profiles and adverse drug reactions, and evaluating relapse rates of dermatophytosis at 12 weeks following treatment completion.

## Materials and methods

This was a prospective, open-label, randomized, parallel-group controlled clinical trial conducted at the Department of Dermatology, All India Institute of Medical Sciences, Raebareli, over 18 months. Patient recruitment occurred over 12 months, followed by six months for data analysis and interpretation of results. Eligible patients aged 15-65 years with clinically suspected dermatophytosis (excluding hair and nail infections) were screened at the outpatient department. Only those meeting the inclusion criteria and providing written informed consent were enrolled. The calculated sample size was 108 patients (54 per group), based on a pooled standard deviation (SD) of 0.7, a clinically significant mean difference of 0.38, 80% power, and a 5% significance level.

A total of 108 patients were enrolled and randomly allocated using a computer-generated sequence into two treatment groups: Group A received conventional itraconazole 100 mg twice daily (C-ITZ, n = 54), and Group B received super bioavailable itraconazole 50 mg twice daily (SB-ITZ, n = 54). During the study period, one patient in Group A and three patients in Group B dropped out for reasons unrelated to adverse events.

Inclusion criteria were dermatophytic infection involving more than 20% of body surface area (BSA), positive KOH mount, body weight >35 kg, and age between 15 and 65 years. Exclusion criteria included pregnancy or lactation, hepatic dysfunction or abnormal liver function tests (LFTs), prior use of systemic antifungals or corticosteroids, recent topical antifungal therapy within four weeks, congestive heart failure, hypersensitivity to itraconazole, and use of concomitant medications known to interact with itraconazole (strong CYP3A4 inhibitors or inducers, QT-prolonging agents, or medications known to reduce absorption).

**Figure 1:**
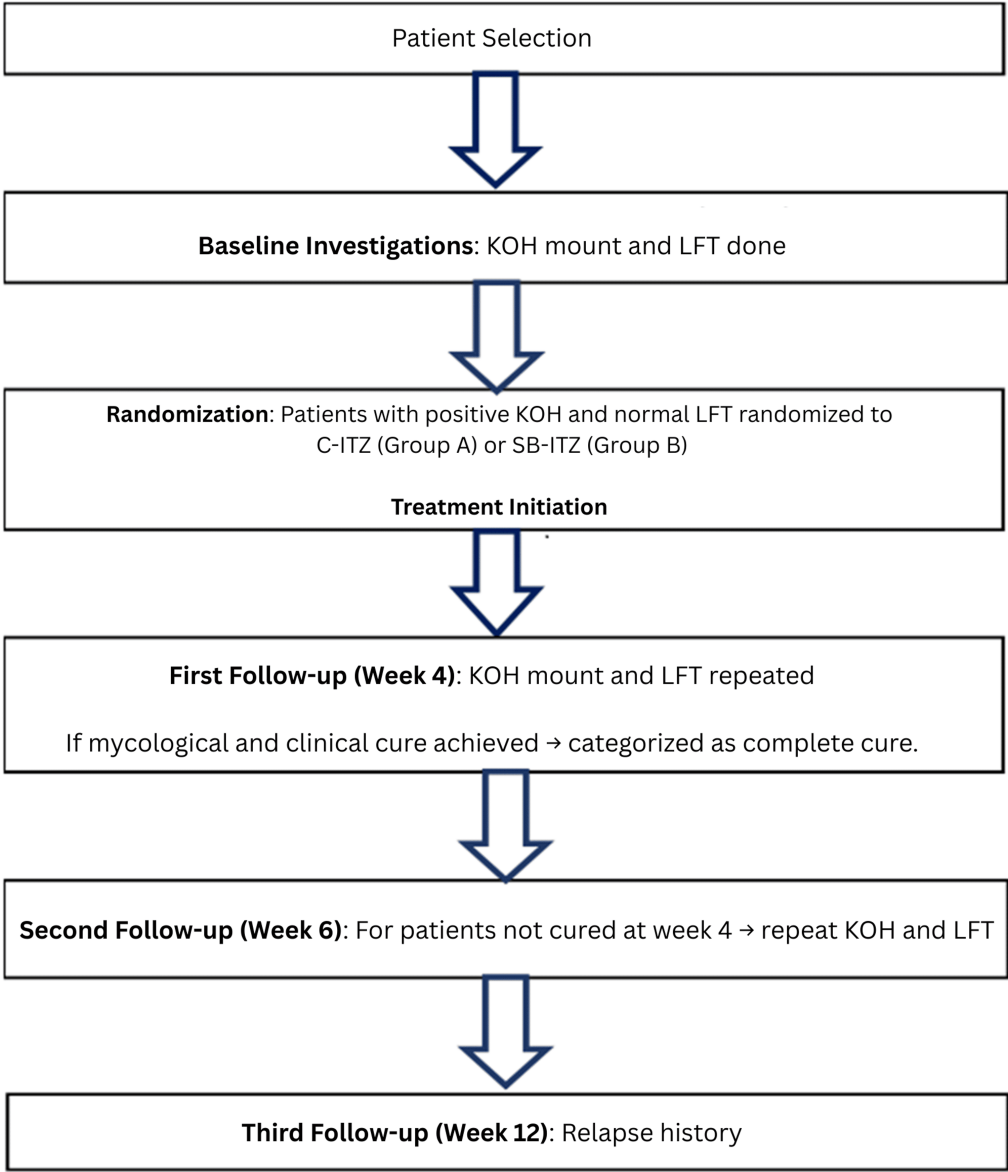
Procedure and data collection C-ITZ: conventional itraconazole, SB-ITZ: super-bioavailable itraconazole, LFT: liver function test

All patients underwent baseline KOH microscopy and LFTs. Adjunctive topical antifungals were prescribed. Both naïve and recalcitrant cases were included; naïve cases were defined as patients without prior treatment, while recalcitrant cases included chronic, resistant, recurrent, or steroid-modified infections with poor or no response to standard therapy. Patients were evaluated at four and six weeks for clinical and mycological outcomes, with LFTs repeated at each visit. Treatment was discontinued if liver enzymes exceeded twice the upper limit of normal. A final follow-up at 12 weeks was conducted to assess relapse (Figure 1).

Outcome assessment

The primary outcome was complete cure at four or six weeks, defined as achievement of both clinical and mycological cure. Clinical cure was assessed using the total symptom score (TSS), which evaluated pruritus, plaques, and erythema/scaling on a 0-3 scale; a score of 0 in all categories indicated clinical cure. Plaques were defined as elevated lesions with measurable thickness and distinct margins caused by active dermatophyte infiltration. Plaques were graded on a scale of 0 to 3 based on thickness, extent, and resolution, with or without post-inflammatory hyperpigmentation (PIH). PIH was not considered the endpoint for this study. The TSS used in this study was developed by the authors based on symptom domains commonly reported in the literature. Because we modified the parameters to reflect the clinical spectrum in our patient population, this exact scoring system has not been previously published or validated. A mycological cure was defined as a negative KOH mount. Secondary outcomes included safety assessment through LFT monitoring, documentation of adverse events, and relapse rates at 12-week post-treatment follow-up.

Statistical analysis

Data were compiled in Microsoft Excel (Microsoft Corp., Redmond, WA, USA) and analyzed using SPSS Statistics (IBM Corp., Armonk, NY). Continuous variables were expressed as mean ± SD, and categorical variables were expressed as percentages. Differences in proportions between groups were tested using a z-test for proportions, as the sample size in each group was large and the data were approximately normally distributed. Comparisons of continuous variables between two independent groups were performed using the independent t-test. Categorical variables with small expected cell counts were compared using Fisher's exact test. A p-value <0.05 was considered statistically significant. The study was conducted in accordance with the Declaration of Helsinki, was approved by the Institutional Ethics Committee of All India Institute of Medical Sciences, Raebareli (approval number: 2024-31-PGTH-7), and was registered with the Clinical Trials Registry of India (CTRI/2024/06/069557). Written informed consent was obtained from all participants.

## Results

Study population

**Figure 2:**
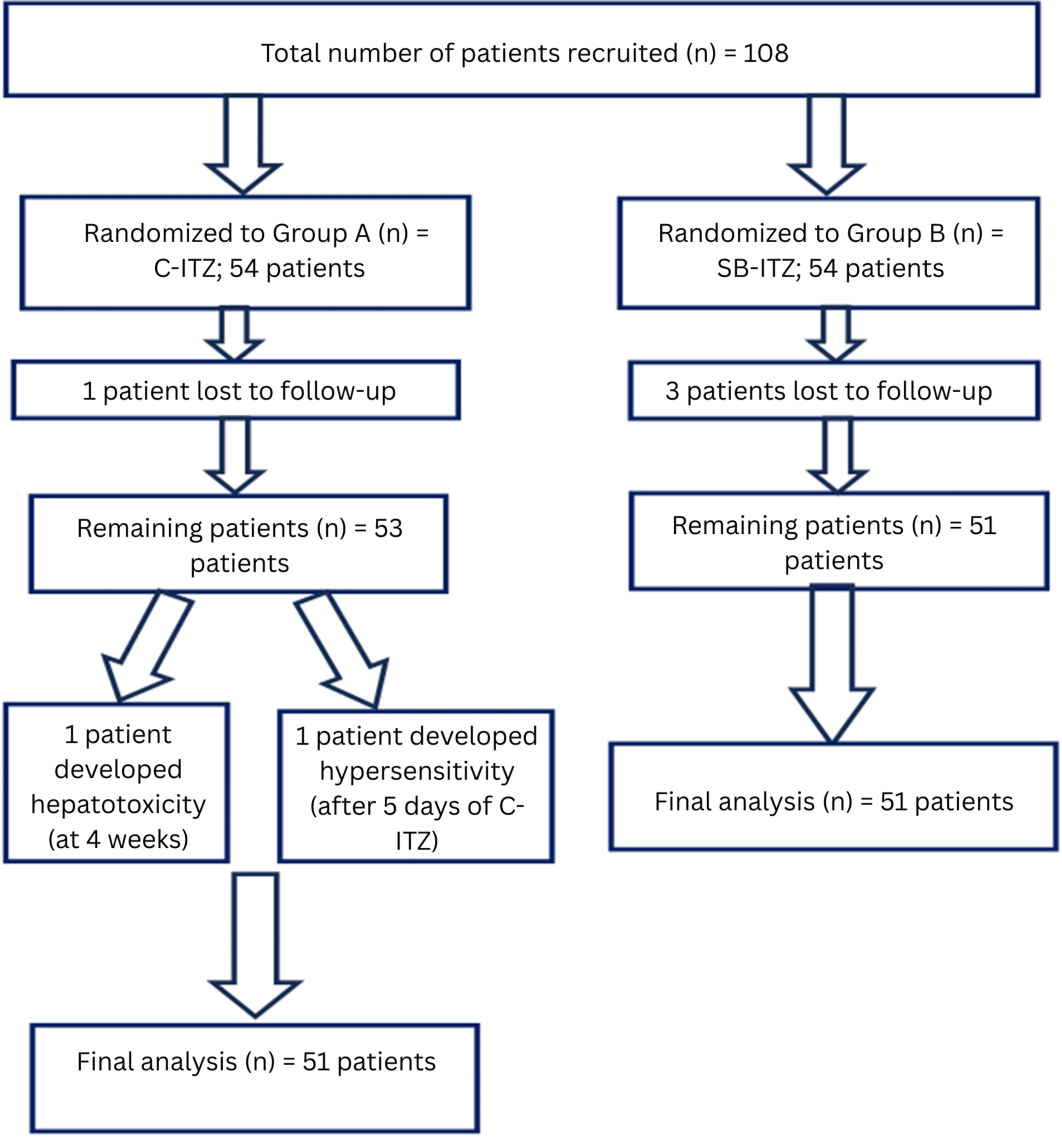
Study design: patient recruitment, randomization, and final analysis C-ITZ: conventional itraconazole, SB-ITZ: super-bioavailable itraconazole

A total of 108 patients were enrolled and randomized in equal numbers to two treatment groups. During the study period, four patients were lost to follow-up, leaving 104 patients who completed the scheduled visits and were included in the baseline demographic and clinical analyses. Subsequently, two patients in Group A discontinued treatment due to adverse events: one developed hepatotoxicity, and one experienced a hypersensitivity reaction to C-ITZ. These patients were excluded from the final efficacy analysis. Consequently, 102 patients (51 in each group) completed the study and were included in the final outcome analysis (Figure 2).

Demographic characteristics

The study population primarily comprised young and middle-aged adults, with most patients falling within the second and fourth decades of life. Very few participants belonged to the older age groups. The mean age was comparable between the two treatment groups, with no clinically significant intergroup difference (Table 1). A slight male predominance was observed overall. However, the gender distribution between the two groups was balanced, and the difference was not statistically significant, indicating adequate baseline comparability (Table 2).

**Table 1:** Distribution of age of patients in Group A and Group B The z-test for proportions was used for categorical variables, whereas the t-test was used to compare means. C-ITZ: conventional itraconazole, SB-ITZ: super-bioavailable itraconazole, SD: standard deviation

Serial number	Age group (years)	Group A (C-ITZ) n (%)	Group B (SB-ITZ) n (%)	Total n (%)	z-/t-value	p-value
1	15-19	4 (7.55)	5 (9.80)	9 (8.65)	1.26	0.21
2	20-24	8 (15.09)	4 (7.84)	12 (11.54)	-1.34	0.18
3	25-29	6 (11.32)	11 (21.57)	17 (16.35)	-0.59	0.56
4	30-34	7 (13.21)	9 (17.65)	16 (15.38)	-1.75	0.08
5	35-39	2 (3.77)	7 (13.73)	9 (8.65)	0.43	0.67
6	40-44	8 (15.09)	6 (11.76)	14 (13.46)	1.59	0.11
7	45-49	8 (15.09)	3 (5.88)	11 (10.58)	0.74	0.46
8	50-54	4 (7.55)	2 (3.92)	6 (5.77)	0.36	0.72
9	55-59	4 (7.55)	3 (5.88)	7 (6.73)	0.59	0.55
1	60-65	2 (3.77)	1 (1.96)	3 (2.88)	1.26	0.21
	Total	53 (100.00)	51 (100.00)	104 (100.00)	-1.34	0.18
	Mean age ± SD	37.13 ± 12.99	34.19 ± 11.41		1.23	0.22

**Table 2:** Distribution of sex of patients in Group A and Group B The z-test for proportion was calculated for categorical variables. M: male, F: female, C-ITZ: conventional itraconazole, SB-ITZ: super-bioavailable itraconazole

Serial number	Sex	Group A (C-ITZ) n (%)	Group B (SB-ITZ) n (%)	Total n (%)	z-value	p-value
1	M	31 (58.49)	26 (50.98)	57 (54.81)	0.77	0.44
2	F	22 (41.51)	25 (49.02)	47 (45.19)	0.77	0.44
3	T	53 (100.00)	51 (100.00)	104 (100.00)	-	-

Housewives represented the largest occupational category across both treatment arms, followed by students, skilled laborers, and individuals engaged in business or other professions. Most patients had attained a school-level education. Overall, the educational profiles of the two groups were comparable and reflected a predominantly semi-educated population.

Clinical history and disease characteristics

A positive family history of dermatophytosis was reported in approximately one-quarter of patients, with a similar proportion noted in both treatment arms. The duration of symptoms varied, with most patients presenting within a few months of disease onset. A subset of patients in both groups had disease duration exceeding six months, including a small number with symptoms persisting for more than one year (Table 3).

**Table 3:** Distribution of duration of symptoms of dermatophytic infection in Group A and Group B C-ITZ: conventional itraconazole, SB-ITZ: super-bioavailable itraconazole

Serial no	Duration (in months)	Group A (C-ITZ)		Group B (SB-ITZ)		Total	
		No. of patients	% of patients	No. of patients	% of patients	No. of patients	% of patients
1	<1 month	4	7.54	3	5.88	7	6.73
2	1-3 months	16	30.19	20	39.21	36	34.61
3	3-6 months	8	15.09	9	17.64	17	16.34
4	6-9 months	14	26.41	6	11.76	20	19.23
5	9-12 months	7	13.20	8	15.68	15	14.42
6	> 1 year	4	7.54	5	9.80	9	8.65
	Total	53	100	51	100	104	100

Pruritus was the most common presenting symptom in both groups, although a small number of patients did not report itching. Typical annular lesions were the predominant morphological pattern observed. Some patients exhibited varied plaque morphology, including atypical or less well-defined lesions. Overall, the symptom profile and lesion characteristics were comparable between the treatment arms.

Recalcitrant cases constituted a larger proportion of the study population than treatment-naïve cases, with nearly equal distribution between the two groups (Table 4). The most frequent clinical presentation involved combined tinea cruris and tinea corporis, followed by isolated tinea corporis. Mixed-site infections were more common than isolated involvement in both groups (Table 5).

**Table 4:** Distribution of category of patients in Group A and Group B C-ITZ: conventional itraconazole, SB-ITZ: super-bioavailable itraconazole

Serial no	Category of patient	Group A (C-ITZ)		Group B (SB-ITZ)		Total	
		No. of patients	% of patients	No. of patients	% of patients	No. of patients	% of patients
1	Naïve	23	43.40	22	43.14	45	43.27
2	Recalcitrant	30	56.60	29	56.86	59	56.73
	Total	53	100	51	100	104	100

**Table 5:** Distribution of type of dermatophytic infection in Group A and Group B based on body region involved C-ITZ: conventional itraconazole, SB-ITZ: super-bioavailable itraconazole

Serial no	Type	Group A (C-ITZ)		Group B (SB-ITZ)		Total	
		No. of patients	% of patients	No. of patients	% of patients	No. of patients	% of patients
1	Tinea corporis	9	16.98	11	21.57	20	19.23
2	Tinea corporis + pedis	1	1.89	0	0	1	0.96
3	Tinea cruris	2	3.77	3	5.88	5	4.81
4	Tinea cruris + corporis	25	47.17	27	52.94	52	50.0
5	Tinea cruris + corporis + faciei	4	7.55	3	5.88	7	6.73
6	Tinea cruris + corporis + mannum	2	3.77	0	0	2	1.92
7	Tinea faciei + barbae + corporis	2	3.77	1	1.96	3	2.88
8	Tinea faciei + cruris	2	1.89	0	0	1	0.96
9	Tinea mannum + pedis + corporis	5	9.43	6	11.76	11	10.58
10	Tinea cruris + corporis + pedis	2	3.77	0	0	2	1.92
	Total	53	100	51	100	104	100

A substantial proportion of patients had a history of tinea, indicating a notable burden of recurrent or relapsing disease in both groups. Prior use of topical medications was common, with topical steroids (46.15%) being the most frequently used therapy. Patients with recent topical use or systemic antifungal/steroid therapy were excluded, ensuring a clean baseline for treatment assessment.

Efficacy outcomes

**Figure 3:**
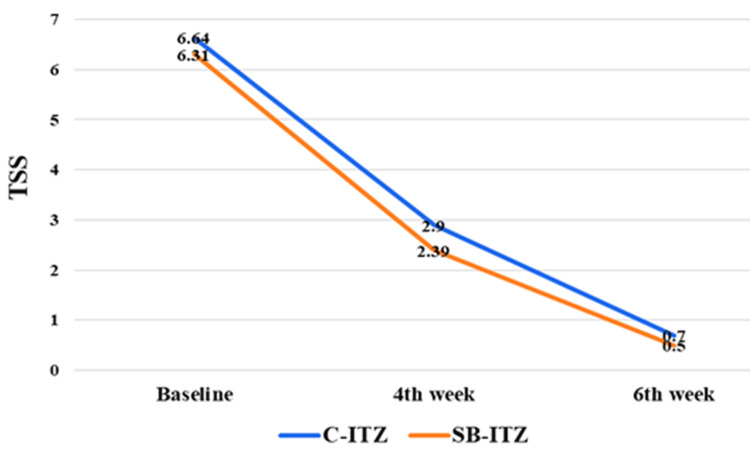
Comparison of TSS between Group A and Group B TSS: total symptom score, C-ITZ: conventional itraconazole, SB-ITZ: super-bioavailable itraconazole

Both C-ITZ and SB-ITZ demonstrated marked clinical improvement over time. BSA involvement and TSS decreased progressively from baseline to six weeks (Table 6, Figure 3). Intergroup differences at all time points were not statistically significant, indicating comparable efficacy between C-ITZ and SB-ITZ. Baseline liver function tests (SGOT, SGPT, and total bilirubin) were comparable between groups. During follow-up, SGOT and SGPT remained stable, with no significant intergroup differences.

**Table 6:** Baseline and follow-up treatment statistics between Group A and Group B An independent t-test was used to compare the mean values. C-ITZ: conventional itraconazole, SB-ITZ: super-bioavailable itraconazole, BSA: body surface area, TSS: total symptom score (maximum TSS: 09)

	Group A (C-ITZ)	Group B (SB-ITZ)	t-value	p-value
	BSA			
Baseline	22.56 ± 9.62	22.27 ± 10.23	0.15	0.88
Follow up (4 weeks)	9.63 ± 6.58	8.56 ± 7.68	0.76	0.45
Follow up (6 weeks)	2.47 ± 3.71	2.15 ± 4.00	0.42	0.67
	TSS			
Baseline	6.64 ± 1.65	6.31 ± 1.84	0.96	0.34
Follow up (4 weeks)	2.90 ± 1.57	2.39 ± 1.47	1.71	0.09
Follow up (6 weeks)	0.70 ± 0.96	0.50 ± 0.88	1.11	0.27

At four weeks, SB-ITZ showed numerically higher rates of clinical and complete cure than C-ITZ, although the differences were not statistically significant (p = 0.07). By six weeks, both clinical and mycological cure rates were higher in the SB-ITZ group, but the differences did not reach statistical significance. Some patients in both groups required continued therapy beyond six weeks (Table 7).

**Table 7:** Cure rates achieved between Group A and Group B The p-value was calculated using the z-test for proportions. C-ITZ: conventional itraconazole, SB-ITZ: super-bioavailable itraconazole

	Group A (C-ITZ) 51 patients	Group B (SB-ITZ) 51 patients	Total	z-value, p-value
Clinical cure				
4 weeks	3 (5.88)	7 (13.73)	10 (9.80)	1.78, 0.07
6 weeks	26 (50.98)	35 (68.63)	61 (59.80)	6.23, 0.401
>6 weeks	22 (43.14)	9 (17.65)	31 (30.39)	8.34, 0.465
Total	51 (100.00)	51 (100.00)	102 (100)	0.0, 1.00
Mycological cure				
4 weeks	9 (17.65)	9 (17.65)	18 (17.65)	0.0, 1.00
6 weeks	28 (54.90)	34 (66.67)	62 (60.78)	1.11, 0.344
>6 weeks	14 (27.45)	8 (15.69)	22 (21.57)	2.12, 0.281
Total	51 (100.00)	51 (100.00)	102 (100)	0.0, 1.00
Complete cure				
4 weeks	3 (5.88)	7 (13.73)	10 (9.80)	1.78, 0.07
6 weeks	26 (50.98)	34 (66.67)	60 (58.82)	5.78, 0.348
>6 weeks	22 (43.14)	10 (19.61)	32 (31.37)	4.38, 0.535
Total	51 (100.00)	51 (100.00)	102 (100)	0.0, 1.00

**Figure 4:**
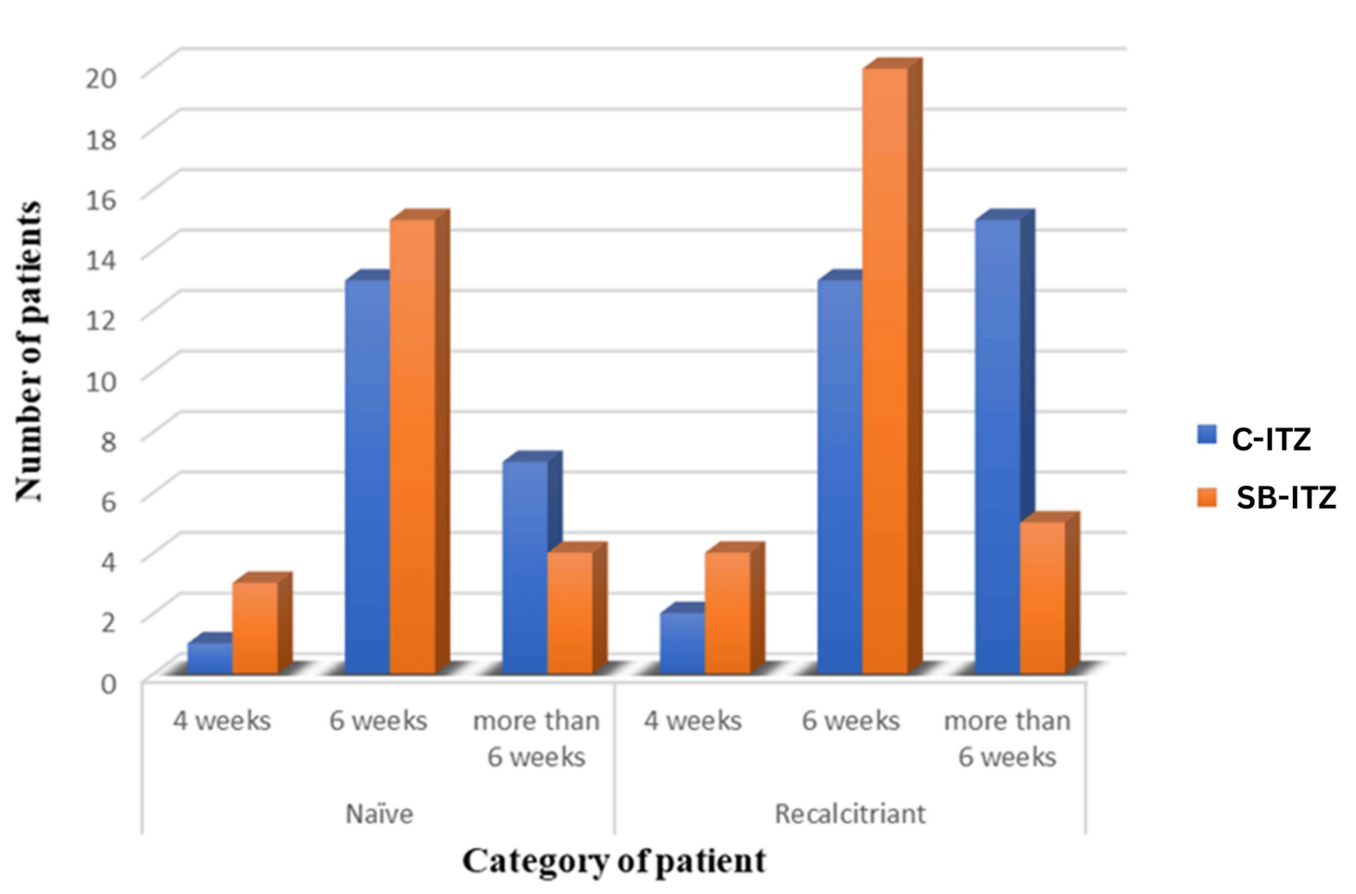
Comparison of clinical cure rates between naïve and recalcitrant patients in Group A and Group B C-ITZ: conventional itraconazole, SB-ITZ: super-bioavailable itraconazole

**Figure 5:**
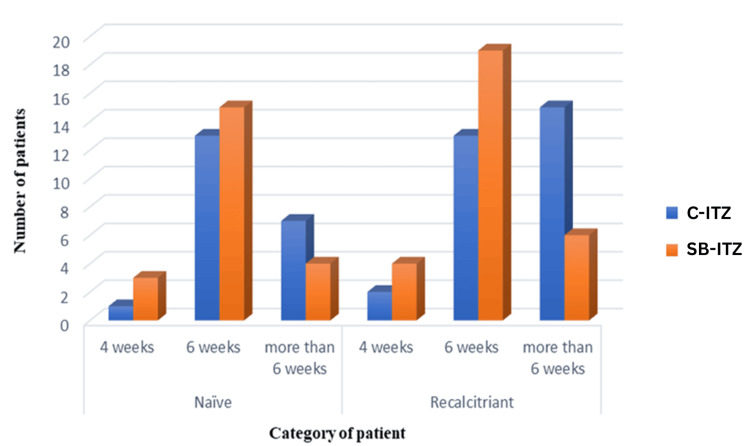
Comparison of mycological cure rates between naïve and recalcitrant patients in Group A and Group B C-ITZ: conventional itraconazole, SB-ITZ: super-bioavailable itraconazole

**Figure 6:**
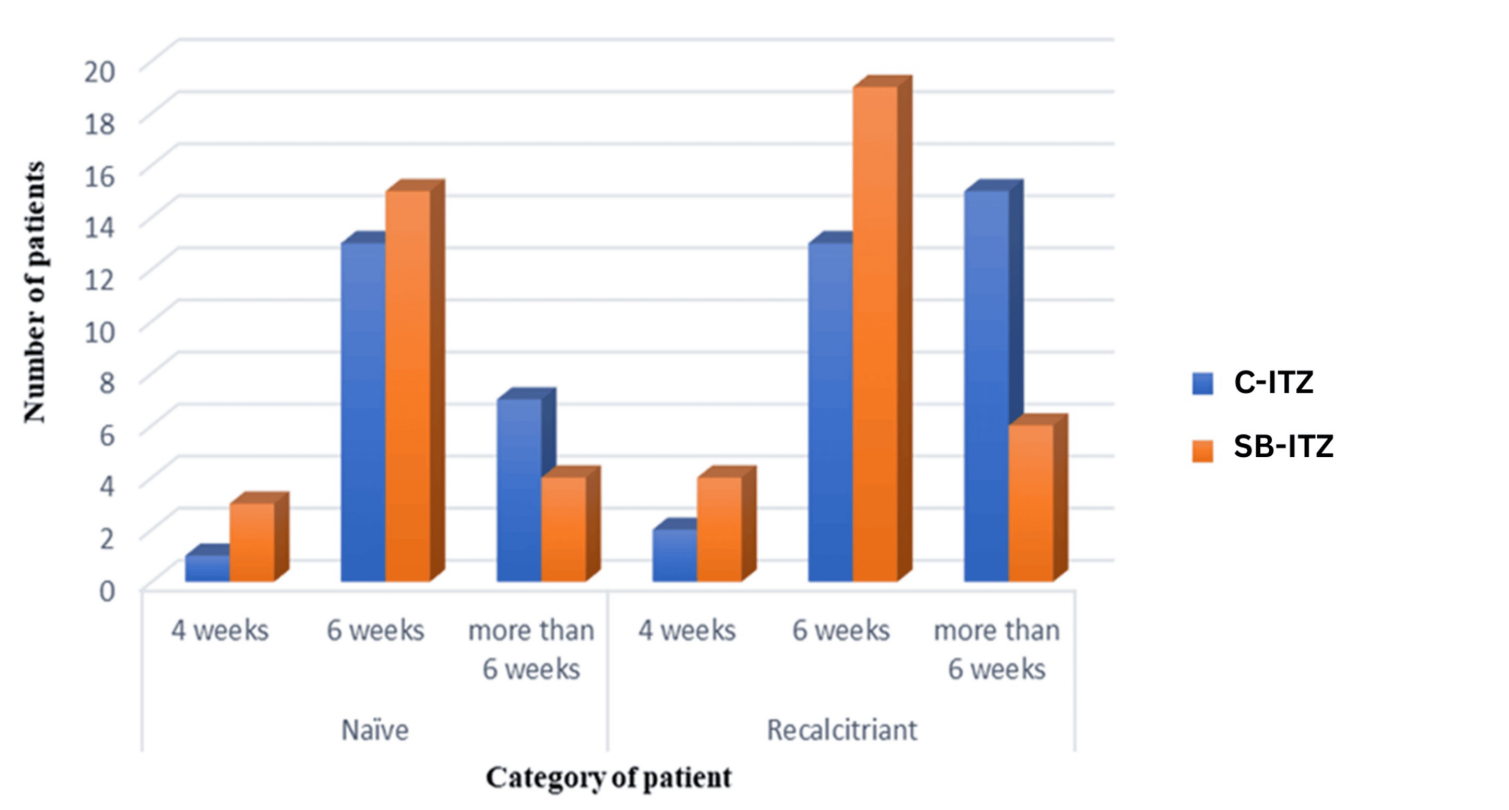
Comparison of complete cure rates between naïve and recalcitrant patients in Group A and Group B C-ITZ: conventional itraconazole, SB-ITZ: super-bioavailable itraconazole

In treatment-naïve patients, clinical, mycological, and complete cure rates were similar between the two groups at all time points, with most achieving a cure by six weeks. In recalcitrant patients, SB-ITZ demonstrated superior efficacy. Clinical cure at six weeks was significantly higher with SB-ITZ (p = 0.029), and fewer patients required prolonged therapy beyond six weeks compared to C-ITZ. Complete cure at six weeks was higher in the SB-ITZ group, approaching significance (p = 0.075), while mycological cure rates remained comparable (Table 8, Figures 4-6).

**Table 8:** Comparison of cure rates achieved between naïve and recalcitrant patients in Group A and Group B The p-value was calculated using the z-test for proportions. C-ITZ: conventional itraconazole, SB-ITZ: super-bioavailable itraconazole

	Group A (C-ITZ)	Group B (SB-ITZ)	Total	z-value, p-value
Naïve patients				
Clinical cure				
4 weeks	1 (4.76)	3 (13.64)	4 (9.30)	1.41, 0.15
6 weeks	13 (61.90)	15 (68.18)	28 (65.11)	0.53, 0.59
>6 weeks	7 (33.33)	4 (18.18)	11 (25.58)	1.27, 0.20
Total	21 (100)	22 (100)	43 (100)	2.57, 0.83
Mycological cure				
4 weeks	6 (28.57)	4 (18.18)	10 (23.25)	0.89, 0.37
6 weeks	12 (57.14)	15 (68.18)	27 (62.79)	0.01, 0.41
>6 weeks	3 (14.29)	3 (13.64)	6 (13.95)	0, 1.00
Total	21 (100)	22 (100)	43 (100)	0.22, 0.83
Complete cure				
4 weeks	1 (4.76)	3 (13.64)	4 (9.30)	1.41, 0.16
6 weeks	13 (61.90)	15 (68.18)	28 (65.11)	0.53, 0.59
>6 weeks	7 (33.33)	4 (18.18)	11 (25.59)	1.28, 0.20
Total	21 (100)	22 (100)	43 (100)	0.21, 0.82
Recalcitrant patients				
Clinical cure				
4 weeks	2 (6.67)	4 (13.79)	6 (10.16)	1.15, 0.250
6 weeks	13 (43.33)	20 (68.97)	33 (55.93)	2.18, 0.029*
>6 weeks	15 (50.0)	5 (17.24)	20 (33.90)	3.18, 0.002*
Total	30 (100)	29 (100)	59 (100)	0.18, 0.850
Mycological cure				
4 weeks	3 (10.0)	5 (17.24)	8 (13.55)	3.14, 0.675
6 weeks	16 (53.33)	19 (65.52)	35 (59.32)	1.12, 0.087
>6 weeks	11 (36.67)	5 (17.24)	16 (27.12)	3.23, 0.660
Total	30 (100)	29 (100)	59 (100)	0.18, 0.85
Complete cure				
4 weeks	2 (6.67)	4 (13.79)	6 (10.16)	2.34, 0.884
6 weeks	13 (43.33)	19 (65.52)	32 (54.24)	3.48, 0.075
>6 weeks	15 (50.0)	6 (20.69)	21 (35.59)	7.56, 0.674
Total	30 (100)	29 (100)	59 (100)	0.18, 0.85

Relapse at the 12-week follow-up occurred in 28.43% of patients overall. While the relapse rate was slightly higher in the C-ITZ group, the difference was not statistically significant (p = 0.43), indicating similar durability of response between the two treatments (Table 9).

**Table 9:** Relapse rates of dermatophytic infection at 12 weeks between Group A and Group B The p-value was calculated using the z-test for proportions. C-ITZ: conventional itraconazole, SB-ITZ: super-bioavailable itraconazole

	Group A (C-ITZ) 51 patients	Group B (SB-ITZ) 51 patients	Total	z-value, p-value
Relapse present at 12 weeks	16 (31.37)	13 (25.49)	29 (28.43)	0.79, 0.43
No relapse	35 (68.63)	38 (74.51)	73 (71.57)	0.496, 0.62
Total	51 (100)	51 (100)	102 (100)	0.0, 1.00

**Figure 7:**
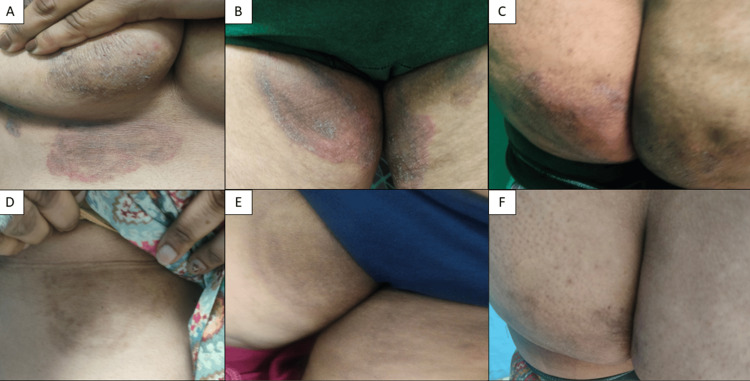
(A–F) Near-complete clearance (>90%) at six weeks in a 25-year-old female with SB-ITZ (recalcitrant case). PIH is noted SB-ITZ: super-bioavailable itraconazole, PIH: post-inflammatory hyperpigmentation

**Figure 8:**
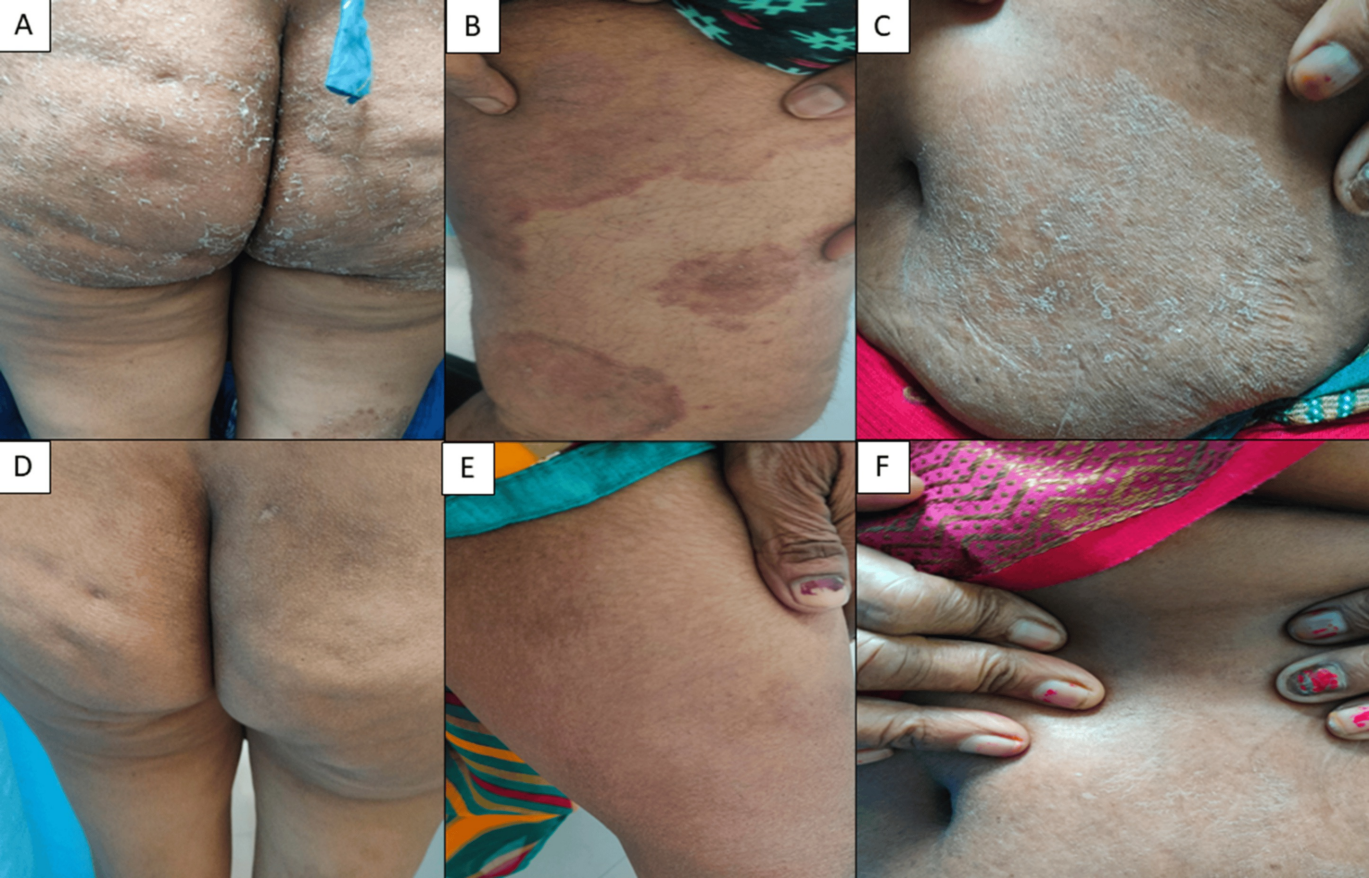
(A-F) Clinical cure seen with C-ITZ at six weeks in a 58-year-old female (recalcitrant case) C-ITZ: conventional itraconazole

**Figure 9:**
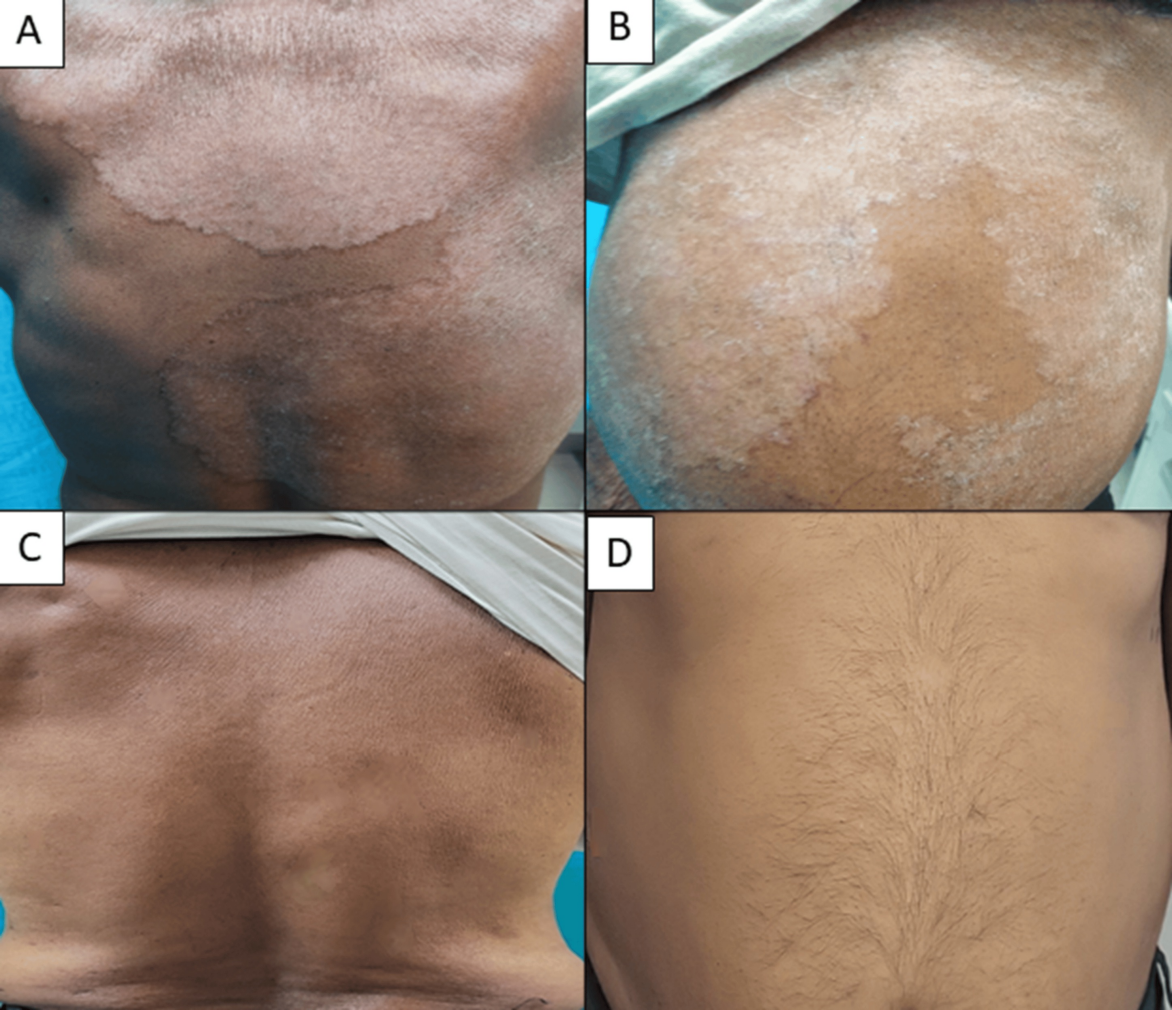
(A-D) Clinical cure seen with SB-ITZ at six weeks in a 61-year male patient with extensive dermatophytosis SB-ITZ: super-bioavailable itraconazole

**Figure 10:**
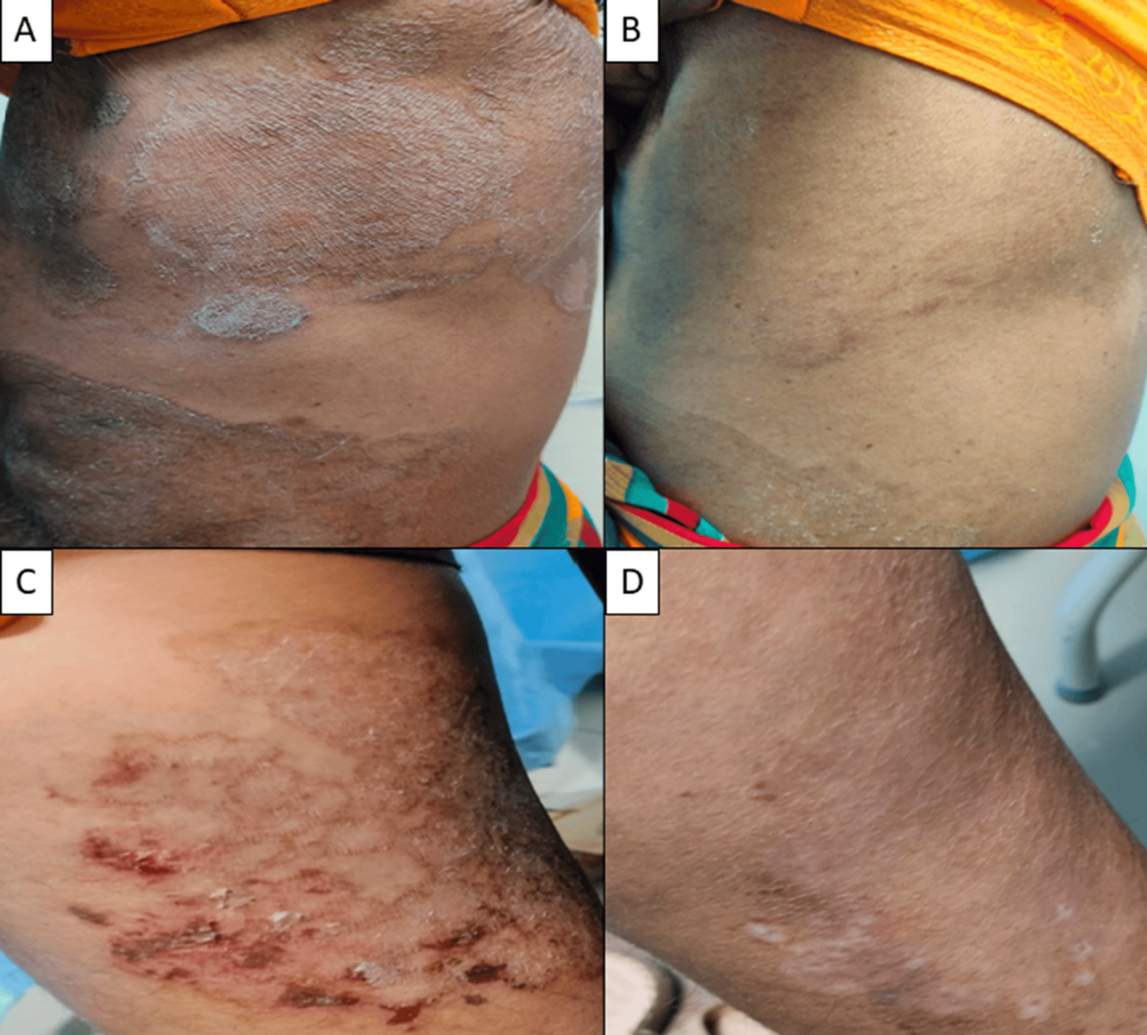
(A-D) Partial resolution in naïve case of tinea corporis requiring more than six weeks duration of treatment with C-ITZ in a 34-year-female C-ITZ: conventional itraconazole

Adverse events were more frequent with C-ITZ, including one case of hepatotoxicity and one hypersensitivity reaction requiring discontinuation. SB-ITZ was associated with fewer adverse effects and better tolerability (Table 10). Clinical photographs showing baseline and post-treatment responses are provided (Figures 7-10).

**Table 10:** Distribution of number of patients reported with various adverse effects between Group A and Group B The p-value was calculated using Fisher's exact test. C-ITZ: conventional itraconazole, SB-ITZ: super-bioavailable itraconazole

	Group A (C-ITZ) 53 patients	Group B (SB-ITZ) 51 patients	Total	p-value (Fisher’s exact test)
Constipation	2 (3.77)	0 (0)	2 (1.92)	0.49
Nausea	1 (1.89)	2 (3.92)	3 (2.88)	1.00
Gastritis	5 (9.43)	2 (3.92)	7 (6.73)	0.26
Deranged LFT (hepatotoxicity)	1 (1.89)	0 (0)	1 (0.96)	1.00
Hypersensitivity reaction	1 (1.89)	0 (0)	1 (0.96)	1.00
No adverse effect	43 (81.13)	47 (92.16)	90 (86.54)	0.15
Total	53 (100)	51 (100)	104 (100)	0.85

## Discussion

India is currently witnessing an unprecedented epidemic of dermatophytosis marked by increasing chronicity, recurrence, extensive BSA involvement, and reduced responsiveness to conventional antifungal therapy. Unlike in earlier decades, when dermatophyte infections were typically localized and short-lived, the current clinical spectrum is characterized by multifocal disease with atypical morphologies, posing significant therapeutic challenges. Several factors have been implicated, including the widespread misuse of topical corticosteroids, poor treatment adherence, inadequate antifungal regimens, and emerging antifungal resistance. Verma et al. have highlighted premature discontinuation of oral antifungals after symptomatic improvement as a major contributor to subclinical persistence and relapse [8].

An important factor contributing to the dermatophytosis epidemic in India is the shifting mycological profile, with recent studies showing increasing predominance of the Trichophyton mentagrophytes/interdigitale complex, particularly in North India, which is associated with greater virulence and reduced susceptibility to azole antifungals [9].

The demographic profile of our study population mirrors trends reported in previous Indian studies. The majority of patients were young or middle-aged adults, with mean ages of 37.13 years in the C-ITZ group and 34.19 years in the SB-ITZ group. Similar age distributions have been reported by Hemdani et al., Radramurthy et al., Mahalaxmi et al., and Pathania et al., who consistently observed that dermatophytosis is most prevalent in the 20-40-year age group [10-13]. Increased physical activity, excessive sweating, the use of occlusive clothing, and frequent exposure to communal environments, such as workplaces and hostels, likely contribute to this susceptibility.

Although males predominated (54.81%) in our study, a rising trend among female patients, particularly housewives, was observed. This observation aligns with findings by Verma et al. and Rudramurthy et al., who reported a narrowing male-to-female ratio in recent years [8,11]. Increased exposure to moist environments during household chores and prolonged occlusion may explain this trend.

Nearly half of our patients reported prior topical corticosteroid use, a finding comparable to that of Pathania et al., who documented steroid misuse in over 50% of patients [13]. Poor hygiene practices, such as sharing towels and footwear and infrequent laundering of clothes, were also commonly observed, reinforcing their role in disease chronicity and recurrence.

Clinically, the most common presentation in our trial was combined tinea corporis et cruris, seen in 50% of patients. This pattern has been consistently reported in recent Indian studies by Shenoy et al., Mohapatra et al., and Mahajan et al., reflecting contiguous spread across adjacent anatomical sites in chronic disease [7,14-15]. Baseline BSA involvement in our study (~22%) was substantially higher than that reported by Shenoy et al. and Mohapatra et al. (~9-10%), highlighting the greater severity of disease in our tertiary-care referral population.

With respect to therapeutic outcomes, both SB-ITZ and C-ITZ demonstrated comparable efficacy across clinical, mycological, and complete-cure rates. Mycological cure rate was achieved earlier than clinical cure, aligning with previous trials. The modest clinical advantage observed with SB-ITZ in recalcitrant cases may be attributable to its improved and more predictable pharmacokinetic profile. However, the absence of significant differences in mycological cure and relapse rates underscores the multifactorial nature of treatment failure in dermatophytosis, including host factors, adherence, hygiene practices, and treatment duration. These findings are consistent with randomized trials by Mohapatra et al. and Shenoy et al., which reported similar efficacy between SB-ITZ and C-ITZ when administered at pharmacologically equivalent doses [14,16].

However, our results differ from those reported by Shenoy et al. and Mahajan et al., who observed significantly higher early cure rates with SB-ITZ [7,15]. This discrepancy may be explained by differences in baseline disease severity, longer disease duration, and the use of SB-ITZ 50 mg rather than the 65 mg formulation employed in some other studies.

A noteworthy observation in our study was the requirement for prolonged therapy beyond six weeks in a substantial proportion of patients, particularly in the C-ITZ group. Relapse rates at 12 weeks remained high and comparable between the two groups, reinforcing observations by Shenoy et al., Ghate et al., and Khurana et al. that relapse prevention depends more on adequate treatment duration, patient compliance, and hygiene practices rather than dose escalation or formulation alone [16-18].

SB-ITZ demonstrated a slightly superior safety and tolerability profile. Serious adverse events requiring discontinuation, namely hepatotoxicity and hypersensitivity, were observed only in the C-ITZ group. These findings are consistent with pharmacokinetic studies by Thompson et al. and other authors, which demonstrated more predictable absorption, reduced interpatient variability, and lower hepatic burden with SB-ITZ [5,19].

An important and unique strength of our study is the inclusion of adolescent patients. Five patients aged between 15 and 18 years were included in the SB-ITZ group, all of whom tolerated the drug well without any adverse effects. This is a notable advantage, as most previously published clinical trials included only patients aged 18 years and above. Our findings therefore suggest that SB-itraconazole may be a safe therapeutic option in older adolescents with recalcitrant dermatophytosis, expanding its potential clinical applicability in this underrepresented age group.

Limitations

The study was limited by its single-center design, relatively small sample size, and lack of blinding, which may restrict the generalizability of the findings. Fungal culture and antifungal susceptibility testing were not performed; mycological cure was assessed using KOH mount alone, which may underestimate persistent infection. The SB-ITZ 50 mg formulation was used rather than the newer 65 mg dose, which may have influenced efficacy in extensive or recalcitrant cases. A longer follow-up would be required to better assess long-term relapse and reinfection.

## Conclusions

SB-ITZ and C-ITZ demonstrated comparable overall efficacy in the management of dermatophytosis. SB-ITZ showed a modest clinical benefit in recalcitrant disease and a more favorable safety profile. These attributes suggest that SB-ITZ could be particularly beneficial in patients with longstanding disease, prior treatment failure, or poor response to conventional therapy. Despite these advantages, optimal treatment outcomes are not solely dependent on the choice of antifungal agent. Factors such as appropriate treatment duration, patient adherence to therapy, and strict hygiene measures are pivotal to achieving sustained clinical and mycological cure. Future multicentric, blinded trials incorporating fungal culture and susceptibility testing are needed to further refine treatment strategies.
